# Variable Response of Norepinephrine Transporter to Traumatic Stress and Relationship to Hyperarousal

**DOI:** 10.3389/fnbeh.2021.725091

**Published:** 2021-09-28

**Authors:** Chiso Nwokafor, Lidia I. Serova, Arax Tanelian, Roxanna J. Nahvi, Esther L. Sabban

**Affiliations:** Department of Biochemistry and Molecular Biology, New York Medical College, Valhalla, NY, United States

**Keywords:** locus coeruleus, gene expression, norepinephrine, epigenetics, hyperarousal

## Abstract

The noradrenergic systems play a key role in stress triggered disorders such as post-traumatic stress disorder (PTSD). We hypothesized that traumatic stress will alter expression of norepinephrine transporter (NET) in locus coeruleus (LC) and its target brain regions which could be related to hyperarousal. Male Sprague-Dawley rats were subjected to single prolonged stress (SPS) and several weeks later the LC was isolated. NET mRNA levels in LC, determined by RT-PCR, displayed variable response with high and low responsive subgroups. In different cohort, acoustic startle response (ASR) was measured 2 weeks after SPS and levels of NET mRNA and protein in LC determined. The high NET responsive subgroup had greater hyperarousal. Nevertheless, NET protein levels, as determined by western blots, were lower than unstressed controls in LC, ventral hippocampus and medial prefrontal cortex and displayed considerable variability. Hypermethylation of specific CpG region in promoter of *SLC6A2* gene, encoding NET, was present in the low, but not high, NET mRNA responsive subgroup. Taken together, the results demonstrate variability in stress elicited changes in NET gene expression and involvement of epigenetic changes. This may underlie mechanisms of susceptibility and resilience to traumatic stress triggered neuropsychiatric symptoms, especially hyperarousal.

## Introduction

Compelling evidence demonstrates that the noradrenergic system plays a key role in stress triggered disorders such as Posttraumatic Stress Disorder (PTSD), depression, and Attention-deficit/hyperactivity Disorder (ADHD; Krystal and Neumeister, 2009; Hendrickson and Raskind, 2016; Kelmendi et al., 2017; Borodovitsyna et al., 2018; Naegeli et al., 2018; Giustino et al., 2019). Both peripheral and central measures of norepinephrine (NE) activity are increased in PTSD patients (Pitman and Orr, 1990; Southwick et al., 1999). NE levels in the cerebral spinal fluid of PTSD patients are elevated and strongly correlated with the severity of the disorder (Geracioti et al., 2001). Furthermore, a polymorphism in the *ADRB2* gene, encoding the β2 adrenoreceptor, interacted with childhood trauma to show greater susceptibility to PTSD (Liberzon et al., 2014).

In the brain, NE is synthesized primarily in the locus coeruleus (LC), which projects widely throughout the brain and receives inputs from a diverse array of brain regions. It provides the sole source of NE in the mammalian forebrain thus mediating a variety of brain functions and behaviors such as arousal, memory acquisition, attention, vigilance, and responses to stress (Aston-Jones et al., 1996; Valentino and Van Bockstaele, 2008; Sara, 2009).

Tyrosine hydroxylase, the rate limiting enzyme in NE biosynthesis, is increased in the LC in rodent stress models including Single Prolonged Stress (SPS) (George et al., 2013; Serova et al., 2013; Sabban et al., 2015). Noradrenergic neuronal signaling is also controlled to a large extent by norepinephrine transporters (NET) by mediating the rapid clearance of NE from the synaptic cleft and maintaining NE storage in the pre-synaptic noradrenergic cells (Torres et al., 2003; Schwartz et al., 2005). After stimulated or spontaneous NE release, NET rapidly clears NE from the synaptic cleft *via* efficient transport system attenuating signaling (Axelrod and Kopin, 1969). NET belongs to the family of sodium chloride neurotransmitter transporters (Amara and Kuhar, 1993) with its concentration in the brain highest in the LC and lower in the cortical and subcortical regions, including the frontal cortex, hippocampus, amygdala, thalamus, and cerebellar cortex (Ordway et al., 1997). It is a target for the action of many antidepressants. NET also plays an important role in presynaptic and postsynaptic homeostasis. For example, mice lacking NET are supersensitive to psychostimulants (Xu et al., 2000). In addition, a polymorphism in the promoter of the *SLC6A2* gene (encoding NET) is associated with panic disorder and anxious arousal symptoms of PTSD (Lee et al., 2005; Pietrzak et al., 2015).

Here, we examine the effect of traumatic stress on NET gene expression in the LC and its relationship to hyperarousal in the single prolonged stress (SPS) model. SPS is a widely used rodent model of traumatic stress that elicits many of the stress-associated maladaptive behaviors relevant to PTSD (Liberzon et al., 1999; Souza et al., 2017; Lisieski et al., 2018). The findings show divergent response in subgroups of the animals and may shed light on mechanism of resilience or susceptibility to stress induced impairments in LC/NE function.

## Materials and Methods

### Animals

All experiments were performed in accordance with the National Institute of Health Guide for Care and Use of Laboratory Animals and approved by Institutional Animal Care and Use Committee at NYMC and the USAMRMC Animal Care and Use Review Office. Male Sprague Dawley rats from Charles River (Wilmington, MA) were 150–160 g on delivery. They were maintained four per cage on a 12 h light/dark cycle at 23 ± 1°C with *ad libitum* access to food and water for 2 weeks to accommodate prior to the experiment. They were then randomly assigned to control or experimental groups.

### Experimental Design

#### Experiment 1

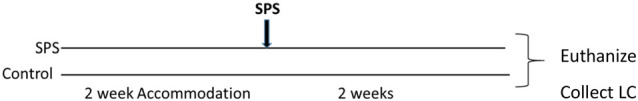



For this experiment, after 2 weeks accommodation, the rats were randomly divided into two groups (*n* = 12 per group). One group was subjected to SPS stressors, and the other control group was handled and weighed at the time of SPS but not subjected to SPS. 2 weeks later the rats were euthanized and the LC was collected from each animal.

#### Experiment 2

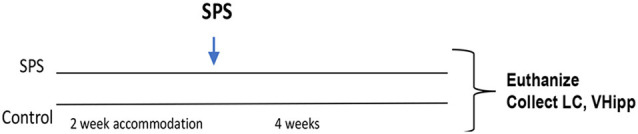



For this experiment, after 2 weeks accommodation, the rats were randomly divided into two groups. One group was subjected to SPS stressors (*n* = 16) and the other control group (*n* = 10) was handled and weighed at the time of SPS but not subjected to SPS. After 4 weeks, the rats were euthanized and the LC and vental hippocampus (vHipp) were collected from each animal.

#### Experiment 3

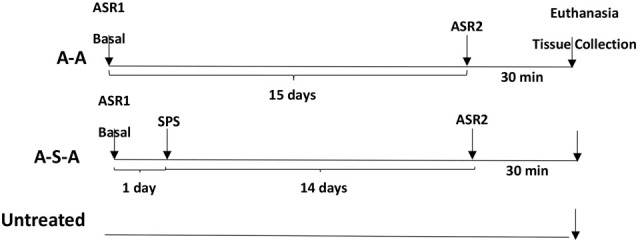



For this experiment, rats were tested for basal acoustic startle (ASR1). They were then randomly assigned divided into two groups one exposed to SPS on the next day (*n* = 11) and one not subjected to SPS (*n* = 8). After 14 days they were tested for ASR again (ASR2) and euthanized 30 min. later. A parallel control group was not exposed neither to ASR nor to SPS. The LC was quick frozen in liquid nitrogen separately from the right and left side of each animal.

#### Experiment 4

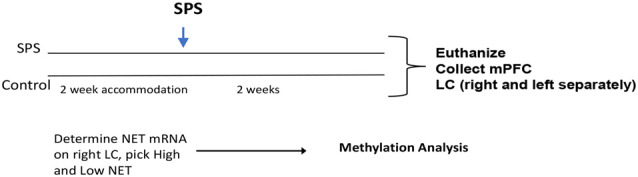



For this experiment, after 2 week accommodation, rats were randomly divided into control or SPS groups (*n* = 16/group). After 2 weeks the rats were euthanized and the medialprefrontal cortex (mPFC) was collected as was the LC, separately from right and left side and frozen into liquid nitrogen. RT-PCR was performed to assess the NET mRNAs in the LC, and the four most extreme from the SPS/high and SPS/low NET were selected for bisulfite methylation analysis of CpG islands in the *SCL6A2* gene promoter.

### Single Prolonged Stress (SPS)

The somewhat modified SPS model of PTSD was performed from 8 AM to 2 PM as previously described (Serova et al., 2013, 2019; Sabban et al., 2015). Briefly, the rats were immobilized by taping the limbs with surgical tape to a metal board which also restricted motion of the head. After 2 h immobilization the animals were subjected to 20 min forced swim individually in a plexiglass cylinder (50 cm high, 24 cm diameter) filled to two-thirds with 24°C water. They were then dried and allowed to recover for 15 min and then exposed to ethyl ether in glass desiccator chamber until loss of consciousness and transferred two per fresh cage. After 7 days undisturbed, they were either tested or kept with normal bedding changes for the remainder of the experiment. Control unstressed animals were from the same cohort and were weighed and transferred two per cage at the time of the SPS.

### Tissue Isolation

Animals were euthanized by decapitation. Brains were dissected using a brain matrix. Sections containing the LC (9.2–10.4 mm posterior to bregma) were dissected. Dissected tissues were placed immediately into cold phospho-buffered saline (PBS). The LC was identified by using reference structures such as the 4th ventricle. A glass pipet with *a* < 1 mm diameter was used to punch out the LC. The medial prefrontal cortex (mPFC) and ventral hippocampus (1.5 to −3.7 mm to bregma and −4.80 to −5.2 mm to bregma, respectively) were isolated. All tissues were transferred to an Eppendorf tube which was flash frozen in liquid nitrogen and stored at −80°C until used.

### Determination of SPS-Triggered Changes in mRNA Levels

RNA Stat (Tel Test, Friendswood, TX) was used to isolate total RNA from the LC. The RNA concentration was determined using NanoDrop 2000 (Thermo Fisher Scientific, Pittsburgh, PA) and 600 ng subjected to reverse transcription with the RevertAid First Strand cDNA Synthesis Kit (Thermo Fisher Scientific) using an oligo dT primer. The cDNA (2 μl) was mixed with 12.5 μl of FastStart Universal SYBR Green Master Rox (Roche Diagnostics, Indianapolis, IN) and 1 μl of one of the following primer sets: NET, (PPRO6785A-200, Qiagen) and GAPDH, (forward 5′-TGGACCACCCAGCCCAGCAAG-3′, reverse 5′-GGCCCCTCCTGTTGTTATGGGGT-3′), to a final volume 25 μl. Reactions were run on a real-time PCR instrument (Applied Biosystems, Carlsbad, CA) and data were analyzed using QuantStudio™ Design and Analysis Software v. 1.4.1. Data normalizing to GAPDH for the same sample, were calculated using the ΔΔCt method (Livak and Schmittgen, 2001) and are presented as a ratio change relative to the mean of the unstressed controls taken as 1.0.

### Western Blots

Total protein from ventral hippocampus, medial prefrontal cortex and locus coeruleus was isolated by homogenization in RIPA buffer. Protein concentration was determined by DC Protein Assay (Bio-Rad, Hercules, CA) with Bio-Tek plate reader. Briefly, 10 μg of total protein were separated on 4%–15% Tris-HCl gradient precast gels (Bio-Rad) and transferred to nitrocellulose membranes (Bio-Rad). After blocking in Tris-buffered saline (TBS) containing 5% dry milk and 0.1% Tween 20 [TBS with Tween 20 (TBST)], membranes were incubated with primary anti-NET (Abcam 41559) overnight at 4°C. After incubation with secondary anti-Rabbit (IRDye 800CW) signal was visualized using the Odyssey Infrared Imaging System (Li-Cor Biosciences, Lincoln, NB) and analyzed using IPLab software (BD Biosciences, San Jose, CA). NET protein levels were normalized to GAPDH with anti-GAPDH (14C10) from Cell Signaling (Danvers, MA).

### Acoustic Startle Response (ASR)

Hyperarousal was assessed by deviation from the basal Acoustic Startle Response (ASR1). ASR was measured as previously described (Nwokafor et al., 2019) in a sound-proof chamber (SR-LAB; San Diego Instruments, San Diego, CA, USA). The piezoelectric accelerometer was calibrated using a stabilimeter for reliable and consistent sensitivity among the chambers. Sound levels within the test chambers were measured with a detachable probe sound level meter to ensure a standardized presentation. Following a 5-min accommodation period with white noise of 68 dB, animals were exposed to 10 repeats of 100 and 115 dB trials for 40 ms (total 20 trials) in random order with inter-trial intervals from 30 to 38 s. Voltage data were collected and transferred to a computer using an automated software package (San Diego Instruments). Enclosures were carefully cleaned with soap and water after each animal.

### Methylation of NET Promoter

Gene-specific DNA methylation was assessed by CD Genomics (Shirley, NY) with next generation sequencing-based bisulfite sequencing PCRs (BSP). In brief, BSP primers were designed using the online MethPrimer software. Genomic DNA (1 μg), isolated from the LC, was converted using the ZYMO EZ DNA, and one twentieth of the elution products were used as templates for PCR amplification. For each sample, BSP products of multiple genes were generated, pooled equally and subjected to adaptor ligation. Barcoded libraries from all samples were sequenced on the Illumina Hiseq platform using paired-end 150 bp strategy.

For the bisulfite sequencing reads of each sample, firstly, adapters and low-quality reads were removed using software Trimmomatic-0.36. After removing the adapter sequences and filtering out the low quality reads, the clean sequencing reads were directly aligned to the target sequences using software Bsmap (v2.73). Methylation levels are defined as the fraction of read counts of “C” in the total read counts of both “C” and “T” for each covered C site. On the basis of such read fraction, methylated cytosine was called using a binomial distribution whereby a probability mass function is calculated for each methylation context. Only those CG covered by at least 200 reads in at least one sample were considered for testing.

### Statistical Analysis

Data were analyzed using GraphPad (Prizm 8, La Jolla, CA, USA). The distributions were analyzed by the Anderson-Darling and Shapiro-Wilk tests for normality. When the distribution failed the test of normality and indicated two subgroups, the values were split with one subgroup containing values more than 2 standard deviations away from the mean of the unstressed controls.

Groups were compared by Student’s t-test or Mann-Whitney non- parametric test to compare two groups, and for more than two groups by one-way ANOVA followed by Tukey’s Multiple Comparison Tests of the Means. Values at *p* ≤ 0.05 were considered significant. For methylation analysis a two-tailed Fisher’s Exact Test was used to identify cytosines that are differentially methylated between two samples or groups. Values were excluded if there was a technical error in ASR test or if sample was degraded.

## Results

### Variable Response of NET Gene Expression in the LC Following SPS

**Figure 1:**
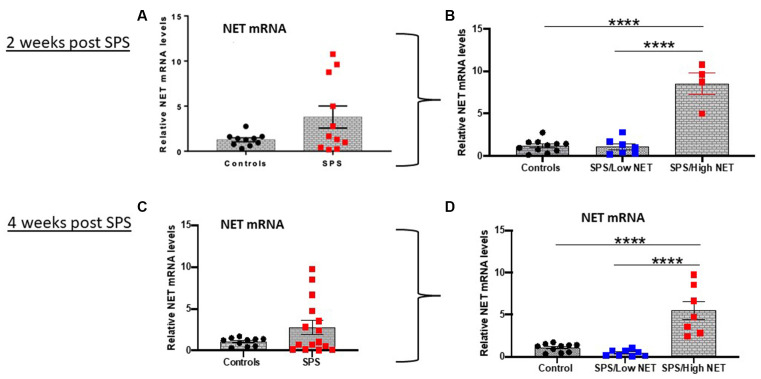
SPS triggered variable response of NET mRNA in LC. Rats (*n* = 12) were exposed to SPS or untreated (*n* = 10) and 2 weeks later they were euthanized and the LC was collected. The relative NET mRNA levels in unstressed controls or 2 weeks after SPS are shown **(A)**. The values in the SPS treated group were divided into low (SPS/LowNET) and high (SPS/High NET) response subgroups **(B)**. The SPS/High subgroup was comprised of NET mRNA levels that were more than 2 standard deviations above the mean of the unstressed controls. In separate experiment (Experiment 2, lower half of figure), rats (*n* = 16) were exposed to SPS or unstressed (*n* = 10). After 4 weeks, the effect of SPS on NET mRNA levels in LC of the SPS group (*n* = 15, one sample lost) compared to controls (*n* = 10) are shown **(C)**. The SPS group was divided into high (*n* = 7) and low responder subgroups (*n* = 8) as above **(D)**. Each dot represents an individual animal, ****p < 0.0001. SPS, single prolonged stress; NET, norepinephrine transporter; LC, locus coeruleus.

The levels of NET mRNA were determined in the LC in two separate cohorts of animals 2 or 4 weeks after exposure to the traumatic stress of SPS (Figure 1). There was an extremely variable response to SPS. Some animals had greatly elevated NET mRNA expression (5–10 fold above controls) while others had NET mRNA levels similar or lower than the unstressed controls. The differences in variance between the control and SPS groups (Figures 1A,C) were significant (*t*_12_= 2.2, *p* < 0.05, *t*_16_= 3.1, *p* < 0.01 at 2 and 4 weeks respectively). While the distribution of the unstressed controls were normal, the SPS treated groups failed the Anderson-Darling and Shapiro-Wilk tests of normality. Therefore, we performed Mann Whitney non-parametric comparison between the control and SPS groups, but it was not significant (*U* = 68, *p* = 0.72).

Subsequently, we divided the SPS-treated animals into two subgroups. The SPS/High NET subgroup had levels of NET mRNA > 2 standard deviations above the mean of the control unstressed group (Alves-Dos-Santos et al., 2020). The other subgroup was termed SPS/Low NET. These subgroups were normally distributed, so the differences were analyzed by one-way ANOVA followed by Tukey comparison of the means. One way ANOVA revealed significance (*F*_(2,19)_ = 57.78; *P* < 0.0001, Figure 1B) and (*F*_(2,22)_ = 23.66; *p* < 0.0001, Figure 1D). Tukey multiple comparisons test showed that the “SPS/High NET” group had NET mRNA levels significantly above the “SPS/Low NET” group as well as above the unstressed controls (*p* < 0.0001, Figures 1B,D).

### Relationship Between Hyperarousal and SPS-Triggered Changes in NET in LC

**Figure 2:**
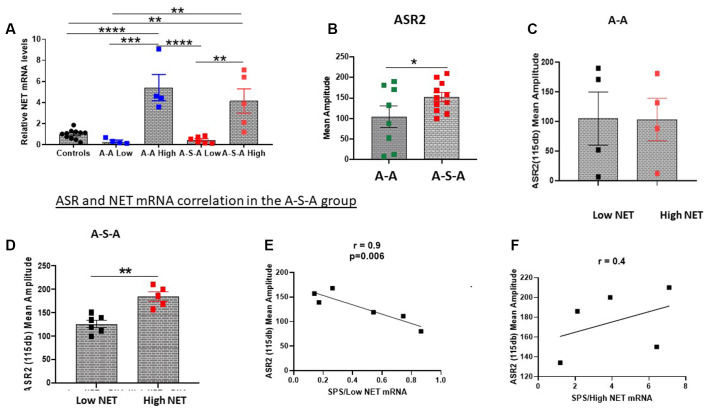
Relationship between changes in ASR and NET gene expression in the LC. NET mRNA expression was determined in groups tested for ASR before and 2 weeks after SPS (A-S-A), or without SPS (A-A) or unstressed controls (Experiment 3). The groups were divided based on two standard deviations from the mean of the controls **(A)**. ASR2 was higher in the A-S-A group compared to the A-A group **(B)**. The associated ASR response of each individual animal to NET mRNA expression is shown for the A-A group **(C)** and A-S-A group **(D)**. The correlation between ASR2 and NET mRNA in the Low NET **(E)** and high NET **(F)** A-S-A subgroup are shown. Each dot represents an individual animal, *p < 0.05, **p < 0.01, ***p < 0.001, ****p < 0.0001. ASR, Acoustic Startle Response.

Since hyperarousal is associated with activation of the NE/LC system (Naegeli et al., 2018) and is elevated in PTSD (O’Donnell et al., 2004) as well as by SPS (Serova et al., 2013; Nwokafor et al., 2019), we examined the relationship between SPS triggered changes in acoustic startle response (ASR) and NET gene expression in the LC (Experiment 3). For this experiment, basal ASR (ASR1) was determined, and one group was exposed to SPS and the other remained unstressed. Two weeks later, ASR was measured again (ASR2) and the animals euthanized. ASR2 was higher in the SPS-treated group (A-S-A group; *t* = 1.858, df = 17, *p* < 0.05, Figure 2B).

After measuring ASR2, the LC was dissected and NET mRNA levels were determined. The groups were divided into subgroups based on NET mRNA levels (Low NET, High NET, Figure 2A). As previously shown, the group subjected to SPS (A-S-A) had higher ASR2 than the group (A-A) not subject to SPS (Figure 2B). The A-A experimental group was similar for both NET subgroups (Figure 2C). In contrast, in the SPS treated group (A-S-A), the high NET subgroup displayed higher amplitude of ASR than the Low NET subgroup (*t* = 4.775, df = 9, *p* < 0.05, Figure 2D). The relationship between ASR2 and relative NET mRNA differed in the two subgroups. In the low NET A-S-A subgroup ASR2 was significantly negatively correlated to relative NET mRNA levels (*r* = −0.9, *p* < 0.01, Figure 2E). In contrast, the high NET A-S-A subgroup there was a positive, but not significant, relationship between NET mRNA levels and ASR2 (Figure 2F).

### Effect of Traumatic Stress on NET Protein Levels in LC and Target Regions

**Figure 3:**
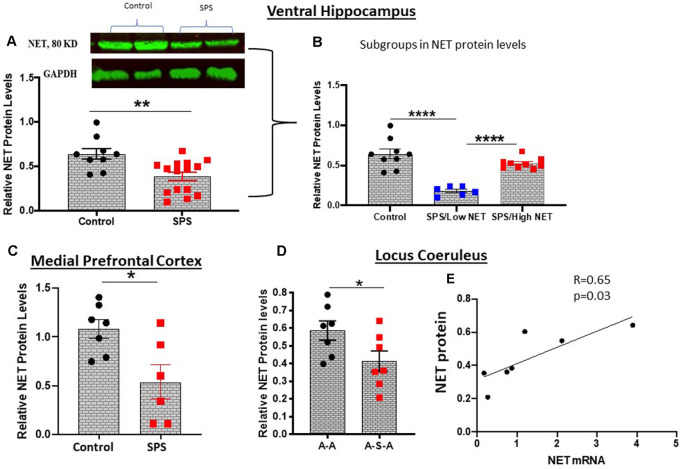
SPS triggered changes in NET protein expression. NET protein levels were evaluated by western blot analysis in the ventral hippocampus 4 weeks after SPS stressors (Experiment 2). A representative western blot and summary data for all the animals **(A)** or when subdivided into high and low NET levels **(B)** are shown. In a different cohort of animals (Experiment 4), NET protein was determined in the medial prefrontal cortex 2 weeks after SPS stressors **(C)**. Relative NET protein levels in LC of the A-A and A-S-A subgroups from Experiment 3 are shown **(D)**. NET protein was correlated to NET mRNA in the A-S-A group (*r* = 0.65, *p* = 0.03) **(E)**. Each dot represents an individual animal, *p < 0.05, **p < 0.01, ****p < 0.0001.

NET is present in the LC as well as in its widespread synaptic terminals. Thus, we determined relative levels of NET protein in the LC, ventral hippocampus and medial prefrontal cortex, key target areas for many of the effects of stress, for example memory and cognition. NET protein levels were examined by Western blot analysis. In the ventral hippocampus 4 weeks after SPS were found to be significantly lower (*t* = 3.3, df = 22, *p* < 0.01) than in the unstressed controls (Figure 3A). There was considerable variability among the animals, which were divided into subgroups (Figure 3B). About half the animals had NET protein levels significantly lower (*F*_(2, 21)_ = 25.50, *p* < 0.0001) than the controls and the other half did not differ from levels in unstressed controls. In another cohort, in the medial prefrontal cortex NET protein levels were significantly lower following SPS than the unstressed controls (*t* = 2.848, df = 11, *p* < 0.05, Figure 3C).

NET protein levels with and without SPS was determined in the LC of animals exposed to ASR (Figure 3). The animals exposed to SPS (A-S-A group) had significantly lower NET protein than in the A-A group not exposed to SPS (*t* = 2.183, df = 12, *p* < 0.05, Figure 3D). Nevertheless, the NET protein levels in the A-S-A was positively correlated (*r* = 0.65, *p* = 0.03) to the NET mRNA levels in the contralateral side of the LC (Figure 3E).

### Methylation of the NET Promoter

**Figure 4:**
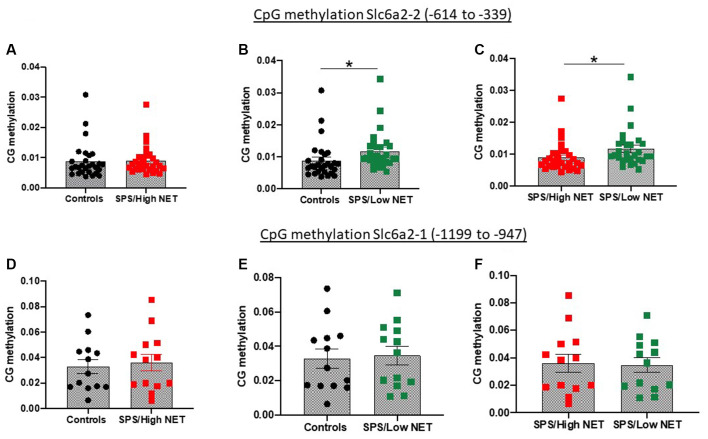
Differences in methylation on two putative CpG islands in the rat promoter of the *SLC6A2* gene (coding for NET) in LC of animals with high **(A,D)** or low NET **(B,E)** gene expression compared to unstressed controls or to each other **(C,F)** in the Slc6a2-2 **(A–C)** or Slc62a2-1 **(D–F)** regions. Animals (*n* = 4 per group) used in the methylation studies were taken from the extremes of animals divided into high and low NET mRNA expression from Experiment 4. Each dot represents a CpG site, *s*p* < 0.05.

Since the animals were significantly diverse in the SPS-elicited NET gene expression, we examined if they might be mediated by differences in methylation of CpG islands in the SCL6A2 promoter. For these experiments, rats were either unstressed or exposed to SPS, and 2 weeks later they were euthanized, and the LC isolated separately from the left and right side. One side of the LC was used to assess NET mRNA levels. Genomic DNA was isolated from the LC from the other side of the brains of the rats which displayed extremely high or low NET mRNA levels after SPS. This was used to determine differences in CpG methylation at two putative CpG regions of the rat SCL6A2 promoter. Methylation of CpG residues in the scl6a2-2 site (−614 to −339) was similar in the unstressed controls in the “SPS/High NET” subgroup (Figure 4A). However, it was elevated in the “SPS/Low NET” subgroup compared to the unstressed controls or to the “SPS/High NET” subgroup (*t* = 1.842, df = 54, *p* < 0.05, Figure 4B) and (*t* = 1.927, df = 54, *p* < 0.05, Figure 4C). In contrast there was no differences in methylation at the scl6a2–1 region at −1,199 to −947 (Figures 4D–F).

## Discussion

To our knowledge, this is the first study to determine changes in NET gene expression in the LC in an animal model of traumatic stress. There was extensive variation in the response of different animals. Weeks after single exposure to SPS, about half the animals had higher NET mRNA expression in the LC and half were similar or lower than in the unstressed controls. These changes in NET gene expression in the LC following SPS were associated with SPS triggered hyperarousal as determined by ASR. The low response subgroup of NET mRNA had hypermethylation of a specific promoter region on the *SCL6A2* gene compared to the high NET response subgroup or to unstressed controls.

Hypermethylation can interfere with the binding of transcription factors therefore methyltransferase activity can result in gene repression. In postural tachycardia syndrome (POTS) patients, changes in NET expression were attributed to increased binding of the repressive MeCP2 regulatory complex, in association with an altered histone modification composition at the promoter region of the *SCL6A2* gene (Bayles et al., 2012). The POTS patients had higher circulating NE as a result of impairment in the clearance of NE by NET from the synapses (Lambert et al., 2008). Other clinical studies revealed genetic variations in the *SLC6A2* gene are associated with neuropsychiatric and/or cardiovascular disease by way of polymorphism in the 3’ end by micro RNA mechanisms (Marques et al., 2017; Hommers et al., 2018).

Epigenetic changes in DNA methylation at a number of loci have been linked to PTSD (Logue et al., 2020). Hypermethylation of a segment of the human *SCL6A2* promoter was observed in ADHD patients compared to healthy controls (Sigurdardottir et al., 2019). In these patients, PET analysis showed negative correlation between methylation of this CpG region and NET distribution in the thalamus, LC and raphe nucleus. Methylation of several sites in this region were associated with lower hyperactivity-impulsivity symptoms (Sigurdardottir et al., 2019), which would be consistent with the findings in our study.

### NET and Hyperarousal

In humans, following traumatic stress hyperarousal develops slowly, and is evident 1–4 months after the traumatic stress (Shalev et al., 2000). Activation of the LC was shown to mediate hyperarousal or hyper-responsiveness in PTSD patients (Naegeli et al., 2018). It is attractive to speculate that the hyperarousal results, at least in part, from the changes in NET in the LC/NE system. In this regard, the NE reuptake inhibitor atomoxetin is approved for treatment of ADHD in children (Yu et al., 2016).

**Figure 5:**
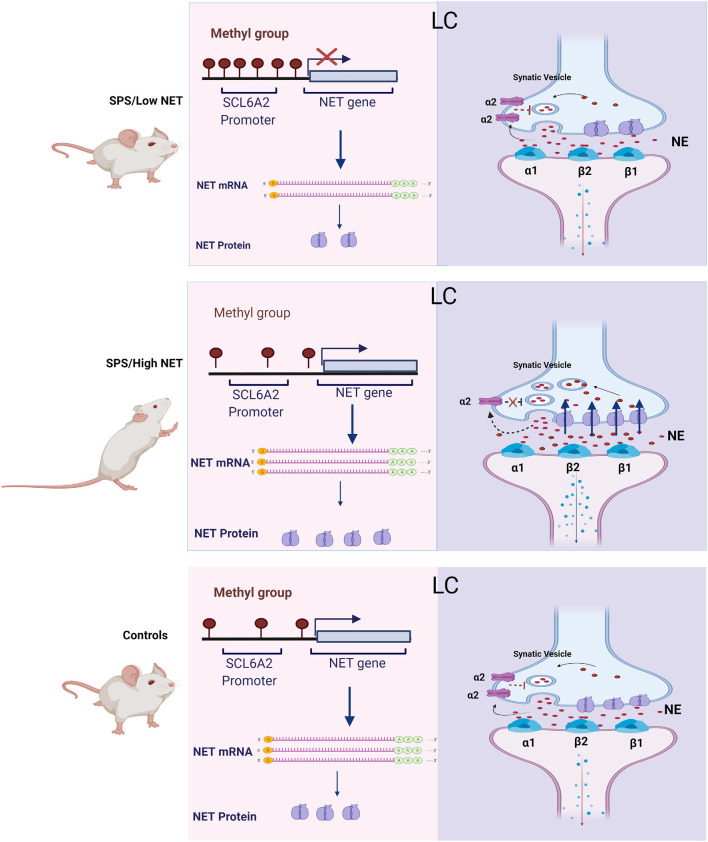
Scheme for changes in NET gene expression and proposed mechanism of differences in activation of the LC in the SPS/LowNET, SPS/HighNET and unstressed control groups. The levels of NE in the synapse are determined by changes in NE synthesis, NET gene expression and auto inhibition at presynaptic cells.

A proposed model for the SPS mediated changes in NET gene expression and their association with noradrenergic transmission is shown in Figure 5. The traumatic stress of SPS was found to elicit a divergent response. Since hypermethylation of the *SCL6A2* gene promoter is not observed in the controls, we speculate that this epigenetic change is triggered by SPS in a subset of the rats. This prevents induction of NET gene expression and of subsequent hyperarousal in SPS/LowNET subgroup.

In the absence of hypermethylation of the *SCL6A2* promoter there is robust induction of NET mRNA expression, which would be expected to lead to enhanced NE clearance. While NET is important in regulating NE levels at the synapse, it is not the only mechanism. Synaptic levels of NE are also influenced by NE synthesis and its release. Additionally, inhibition of presynaptic receptors can increase synaptic levels of NE. The α2 and Y2R presynaptic receptors negatively affect the release of NE. In the high NET responsive group, SPS may trigger reduced auto inhibition of firing, enhanced LC activation with subsequent increased NE signaling in target regions and elevated hyperarousal in this situation.

Inhibition of NET by antidepressants has been shown to change the density and function of pre- and postsynaptic adrenergic receptors. Furthermore, NET-deficient (NET-KO) mice behave like antidepressant-treated mice with upregulation of mRNAs encoding the α2A- and α2C- adrenergic receptors in the brainstem (Gilsbach et al., 2006).

This changes in NET expression observed in this study may not only affect hyperarousal, but perhaps also susceptibility to drugs of abuse. Cocaine not only binds to the dopamine transporter but also to NET. The anxiety-like behavior that is observed during cocaine abstinence and the stress-induced relapse to cocaine use may be mediated by the effects of cocaine on noradrenergic circuits originating from brainstem nuclei [e.g., locus coeruleus (LC)]. Indeed, NET knockout mice are hyper-responsive to locomotor stimulation by cocaine or amphetamine (Xu et al., 2000).

### Changes in Stress-Triggered NET Gene and Protein Expression

Previously, chronic social stress was reported to increase NET mRNA and protein levels in the rodent LC and projection regions of the hippocampus, frontal cortex and amygdala which could lead to reduced NE at their synapses (Chen et al., 2012). It has been proposed that the induction of NET is mediated by activation of the hypothalamic pituitary adrenal (HPA) axis. Chronic treatment with corticosterone for 21 days appeared to mimic this effect and increased NET mRNA and protein in the LC, as well as NET protein levels in the hippocampus, frontal cortex and amygdala (Fan et al., 2014).

In this study, although SPS elicited a large rise in NET mRNA levels in the LC in about half the animals after 2 weeks, NET protein levels were lower than in the comparable controls not subjected to SPS. Nevertheless, the reduction was less extensive in the animals with elevated NET mRNA levels and NET protein levels were correlated with the change in NET mRNA levels. Perhaps, the increased NET mRNA levels are an attempt to compensate for the reduced NET protein remaining in the LC.

Unexpectedly, despite the large elevation of NET mRNA in the LC in many of the animals, NET protein levels in target region of the ventral hippocampus were not elevated and actually lower than the unstressed controls in about half the SPS-treated animals. NE from the LC is involved in modulating the encoding, consolidation, retrieval and reversal of hippocampal based memory (Hansen, 2017). NE in the hippocampus was shown to regulate transcriptional control of long-term plasticity to gate the endurance of memory storage. As a result, the LC/NE system is key in orchestrating longevity of hippocampal-dependent memory. Thus, reduced NET in the hippocampus keeps high NE in the synapse which could enhance memory consolidation of the trauma resulting in an increased chance of re-experiencing the traumatic event when a reminder is present. Enhancement of NE signaling can bidirectionally modulate mPFC functions. The LC input to the mPFC plays an essential cognitive functions, such as cognitive flexibility and attentional set shifting (Morilak et al., 2005; Sara and Bouret, 2012; Jett and Morilak, 2013). Future studies should determine if traumatic stress also alters NET expression in other noradrenergic locations in the brain, such as the NTS, a key regulator of the hypothalamus, and its effects as well on NET expression in peripheral noradrenergic sympathetic nerves.

The discrepancy between the changes in mRNA in the LC and protein in the hippocampus and mPFC might reflect a lag between transcription and translation or transport of the protein. Also, SPS might be altering the turnover rate of the protein. Further studies with more projection areas and a more detailed time course could help to clarify this. However, NET activity can be regulated not only by its protein levels, but also by its plasmalemmal distribution. Chronic cold stress increased the proportion of NET on the plasma membrane in the rat prefrontal cortex (Miner et al., 2006). NET is also regulated by several extracellular and intracellular signaling molecules. Post translationally, NET is regulated by glycosylation and phosphorylation (Torres et al., 2003; Mandela and Ordway, 2006). The carbohydrate units of glycoproteins are involved in controlling protein folding, stabilizing protein conformation, protecting against proteolysis, as well as regulating intracellular and surface trafficking (Lis and Sharon, 1993; Melikian et al., 1996; Nguyen and Amara, 1996). In addition, protein kinase C can phosphorylate both serine and threonine residues in rat and human NET, resulting in transporter phosphorylation and down-regulation (Jayanthi et al., 2006).

Although NET heterozygous knockout mice display approximately 50% reduction in NET protein levels, this reduction in NET did not lead to a deficit in NET activity either in hippocampal or cortical synaptosomes due to transport compensatory mechanisms such as serotonin, dopamine or organic cation transporters (Fentress et al., 2013). Future studies could also measure, the transport of NET into synaptosomes, and isolation of membrane fractions/biotinylation of cell-surface proteins.

Overall, the results of this study demonstrate the importance and complexity of stress elicited changes in the NET gene expression. Further work is needed to completely elucidate the significance of variable response of NET to traumatic stress in LC mediated functions. The findings could shed new light on underlying mechanisms for susceptibility and resilience to traumatic stress triggered neuropsychiatric symptoms and especially hyperarousal.

## Data Availability Statement

The raw data supporting the conclusions of this article will be made available by the authors, without undue reservation.

## Ethics Statement

The animal study was reviewed and approved by Institutional Animal Care and Use Committee at New York Medical College and the USAMRMC Animal Care and Use Review Office.

## Author Contributions

CN performed the majority of the experiments, data analysis, and writing the manuscript. LS played a crucial role in planning, conducting, and interpreting the experiments. AT and RN participated in performance and analysis of the experiments. ES planned the study, and was involved in interpretation of the results, and in writing the manuscript. All authors contributed to the article and approved the submitted version.

## Conflict of Interest

The authors declare that the research was conducted in the absence of any commercial or financial relationships that could be construed as a potential conflict of interest.

## Publisher’s Note

All claims expressed in this article are solely those of the authors and do not necessarily represent those of their affiliated organizations, or those of the publisher, the editors and the reviewers. Any product that may be evaluated in this article, or claim that may be made by its manufacturer, is not guaranteed or endorsed by the publisher.
